# Fever as an Initial Manifestation of Enthesitis-Related Arthritis Subtype of Juvenile Idiopathic Arthritis: Retrospective Study

**DOI:** 10.1371/journal.pone.0128979

**Published:** 2015-06-01

**Authors:** Ruru Guo, Lanfang Cao, Xianming Kong, Xuesong Liu, Haiyan Xue, Lijuan Shen, Xiaoli Li

**Affiliations:** 1 Department of Pediatrics, Renji Hospital, School of Medicine, Shanghai Jiaotong University, Shanghai, P. R. China; 2 Department of Ultrasound, Renji Hospital, School of Medicine, Shanghai Jiaotong University, Shanghai, P. R. China; University of East London, UNITED KINGDOM

## Abstract

**Objective:**

We wished to determine the prevalence of fever as one of the first symptoms of the enthesitis-related arthritis (ERA) subtype of juvenile idiopathic arthritis. Also, we wished to ascertain if ERA patients with fever at disease onset differed from those without fever.

**Methods:**

Consecutive cases of ERA were diagnosed and followed in a retrospective observational study from 1998 to 2013. Information about clinical/laboratory data, medications, magnetic resonance imaging (MRI), and disease activity during the study period was also recorded.

**Results:**

A total of 146 consecutive ERA patients were assessed. Among them, 52 patients (35.6%) had fever as one of the first symptoms at disease onset. Compared with ERA patients without fever at disease onset, patients with fever had significantly more painful joints (3.5 *vs*. 2.8), more swollen joints (1.1 *vs*. 0.8), and more enthesitis (1.0 *vs*. 0.4) (p<0.05 for all comparisons). Patients with fever had significantly higher mean values of erythrocyte sedimentation rate, C-reactive protein, platelet count, and child health assessment questionnaire (CHAQ) scores (40.8 *vs*. 26.4 mm/h; 20.7 *vs*. 9.7 mg/dL; 353.2×10^9^/L *vs*. 275.6×109/L; 1.0 *vs*. 0.8, respectively; all p<0.05). During two-year follow-up, CHAQ score, number of flares, as well as the number of patients treated with oral non-steroidal anti-inflammatory drugs, corticosteroids and combination therapy with disease-modifying anti-rheumatic drugs, were significantly higher in ERA patients with fever.

**Conclusions:**

Fever was a frequent manifestation of ERA. ERA patients with fever had more active disease at disease onset and poorer outcomes than ERA patients without fever.

## Introduction

Enthesitis-related arthritis (ERA) is a chronic, inflammatory disease. ERA accounts for ≈7–37% of cases of juvenile idiopathic arthritis (JIA) [1, 2]. Studies from India and China have shown ERA to be the most prevalent category of JIA [1]. Patients with ERA have a clinical picture similar to that of individuals meeting the traditional definitions of juvenile spondyloarthropathies, including juvenile ankylosing spondylitis, seronegative enthesopathy, arthropathy syndrome, and undifferentiated juvenile spondyloarthropathy (SpA) [3].

ERA is an HLA-B27-associated type of pediatric inflammatory arthritis characterized by involvement of entheses, the axial skeleton and peripheral joints, and is associated with a poorer prognosis compared with other categories of JIA [2, 4, 5]. Data from studies carried out in various populations and clinical settings suggest an increase in the prevalence and incidence of juvenile-onset SpA in recent years [6].

In general, ERA patients manifest insidious-onset, slight/moderate intermittent symptoms in multiple joints and entheses, and poor physical status [7]. During episodes of disease activity, systemic features occur in 5–10% of ERA patients and comprise: fever; weight loss; weakness and atrophy in muscles; fatigue; enlargement of lymph nodes; leukocytosis; anemia [8]. ERA patients with fever as one of the first symptoms at disease onset are often diagnosed as having septic arthritis or systemic onset juvenile idiopathic arthritis (soJIA). These circumstances can yield mistreatment in early stages of the disease and late referral to rheumatologists.

Recognition and the differential diagnosis may be important problems in early ERA, particularly if children present with non-specific complaints (e.g., fever, pains, tiredness). Hence, the aim of the present study was to describe the clinical manifestations, treatment course, as well as the laboratory and imaging features of ERA patients with fever. Also, we wished to ascertain if ERA patients with fever at disease onset differed from those without fever.

## Materials and Methods

The medical records of all children who were hospitalized in the Pediatric Department of this hospital between 1998 and 2013 were studied. One hundred and forty-six enthesitis-related arthritis (ERA) patients were diagnosed by pediatric rheumatologists and followed for two years, from a pool of 290 patients having chronic joint pain. The diagnosis of ERA was made using the criteria of the International League of Associations for Rheumatology (ILAR), published in 2004 [9]. Fever was defined as an axillary body temperature 37.5°C and higher and classified into irregular fever, intermittent fever and remittent fever [10]. Based on the height of body temperature, ERA patients can be classified into fever group and non-fever group. Exclusion criteria for this study included infectious disease, leukemia, lymphoma and other connective tissue diseases. The study was conducted according to the Declaration of Helsinki and ethics committee approval for this study was obtained at Renji Hospital, School of Medicine, Shanghai Jiaotong University. Guardians on behalf of the children gave written informed consent according to the guidelines of the ethical research committee of Renji Hospital, School of Medicine, Shanghai Jiaotong University prior to their inclusion in the present study.

### Study design

Detailed clinical profiles and investigation results were retrospectively reviewed. The following variables were entered into a customized database.

#### Demographic variables

(1) History of HLA-B27-related disease in a first-degree relative, including ankylosing spondylitis, ERA, inflammatory bowel disease, reactive arthritis (Reiter’s syndrome), and acute anterior uveitis; any individual or family history of atopy (extending to first and second degree relatives). Psoriasis was excluded since it is a mandatory exclusion criterion for ERA diagnosis if present in patient and/or in a first-degree parent; (2) patient’s sex; (3) age at onset of disease; Disease onset was defined as the data when the child, according to anamnestic information, fulfilled the criteria of active arthritis or onset of systemic features, not necessarily conformed by a doctor; (4) age at diagnosis of ERA.

#### Clinical variables

(1) Disease duration; (2) assessment of the number and type of affected joints (number of painful joints, number of swollen/effused joints, number of joints with limitation of motion [LOM]). Joints involvement was defined as joint swelling or limitation in the range of joint movement with joint pain or tenderness, which persists for at least 6 weeks; (3) assessment of the number and type of affected entheses. Enthesitis was defined as discretely localized tenderness at the point of insertion of ligaments, tendons, joint capsules, or fascia to bone [2]; (4) inflammatory back pain, defined according to the Assessment of Spondyloarthritis International Society (ASAS) expert criteria [11]; (5) tenderness of sacroiliac (SI) joints, compression of pelvis, distraction of the SI joints by Patrick’s test [2]; (6) the height of body temperature, fever pattern, and administration of antibiotics due to suspected infection.

#### All other variables

(1) Laboratory variables: erythrocyte sedimentation rate (ESR), C-reactive protein (CRP), leukocyte, platelet, hemoglobin, bone marrow smear, antinuclear antibodies (ANA), rheumatoid factor (RF) and presence of the HLA-B27 allele; (2) Therapy administered at onset and throughout the disease course; (3) Disease activity measures, according to the American College of Rheumatology pediatric criteria [12]; (4) Disease remission indices, according to the preliminary criteria for clinical remission in JIA [13], including no active enthesitis; Occurrence of relapse was registered if there is a presence of remission, followed by new periods of activity; (5) assessment of functional health status, measured by Childhood Health Assessment Questionnaire (CHAQ) [14]; (6) a general examination was also complemented by global assessments (including the physician global assessment [PGA] of disease activity, and parent’s global assessment of the child’s overall well-being using a 10 cm horizontal visual analogue scale [VAS]) [15]; (7) a diagnosis of atopy was made in the context of a positive serum specific IgE concentration of >0.70 kU/L, a positive skin prick test (SPT) results, or an individual and family history of atopy (at least two of the three criteria positives being regarded as atopy) [16].

#### Imaging data

Ultrasound (gray-scale [GS] and power Doppler [PD]) and magnetic resonance imaging (MRI) of joint involvement or enthesitis were obtained. Abnormalities by ultrasound (US) were considered suggestive of enthesitis [17]: increased thickness and/or hypoechogenicity (compared to the other side), structural abnormalities (calcifications, enthesophytes, and/or erosions), and vascularization at the entheses insertion into cortical bone, taken to indicate inflammation. The utility of MRI early in disease is to identify active inflammation of involved joints before radiographs are abnormal. As reported [4], early changes include synovitis, joint effusion and bone marrow edema (BME). MRI protocol: all examinations were performed on a 16-channel 3.0 Tesla MRI scanner (Ingenia, Philips Medical Systems, The Netherlands). Localizer was taken in axial and sagittal planes after making proper positioning of the patient. The MRI protocol consisted of sagittal T1W, sagittal T2W, axial T1W and axial T2W sequences. Coronal SPAIR images were acquired. MR sequences were evaluation by two experienced radiologists.

### Statistical analysis

Categorical data were expressed by counts or percentages, and compared between different groups using the chi-squared test or continuity correction as appropriate. The continuous variables were presented as the mean (standard deviation), and were analyzed by Student’s t-test or the Mann-Whitney U-test as appropriate. A p-value of < 0.05 was considered statistically significant. All analyses were performed using the SPSS software version 13.0 (SPSS Inc.; Chicago, IL, USA).

## Results

### Characteristics of patients

Of the 146 included children classified as having ERA, 52 (35.6%) had fever as one of the first symptoms at disease onset. A total of 119 boys and 27 girls had a mean age at the diagnosis of 10.3 years. Boys outnumbered girls at a ratio of 4.4:1. A total of 86 patients (77 male, 9 female; 58.9%) were positive for HLA-B27. A positive family history for HLA-B27-related diseases was present in 40/146 subjects (27.4%). A total of 38.4% of patients were positive for inhaled allergy, 41.8% for food allergy, and 39.0% for SPT. Upon comparison with the non-fever group, the fever group had a significantly higher: mean parent global assessment of child’s overall well-being VAS score; number of painful joints; number of entheses with enthesitis; tender entheseal score; frequency of positivity of Patrick’s test; parent global assessment of child’s pain VAS score (all p<0.05, Table 1). In particular, disease severity measured by the Childhood Health Assessment Questionnaire (CHAQ) score in the fever group was significantly higher than the non-fever group at disease onset and the end of two-year follow-up (p = 0.010). Time between symptom onset and the diagnosis in the fever group was significantly longer than that in the non-fever group (p = 0.040).

**Table 1:** Demographics and characteristic of ERA at onset.

Characteristic	Non-fever (n = 94)	Fever (n = 52)	p
Age at diagnosis†, years	10.2 (2.8)	10.4 (2.8)	0.674
Gender boys‡, n (%)	72 (76.6)	47 (90.4)	0.040*
Time between onset of symptoms and diagnosis†, months	4.1 (4.1)	6.7 (6.8)	0.005*
Individual history of inhaled allergy‡, n (%)	32 (34.0)	24 (46.2)	0.150
Individual history of food allergy‡, n (%)	34 (36.2)	27 (51.9)	0.065
SPT, positive‡, n (%)	33 (35.1)	24 (46.2)	0.190
Family history of atopy‡, n (%)	42 (44.7)	30 (57.7)	0.132
A family history positive for HLA-B27 related diseases‡, n(%)	24 (25.5)	16 (30.8)	0.497
HLA-B27 presence‡, n (%)	56 (59.6)	30 (57.7)	0.825
Uveitis§, n (%)	7 (7.4)	4 (7.7)	1.000
**Disease characteristics** †			
PGA of disease activity VAS	4.4 (1.2)	4.2 (1.1)	0.466
Parent global assessment of child’s overall well-being VAS	3.8 (1.5)	4.4 (1.2)	0.025*
No. of active joints	4.0 (2.3)	4.3 (2.2)	0.442
No. of joints with LOM	1.1 (1.4)	1.0 (1.1)	0.672
No. of painful joints	2.8 (1.7)	3.5(1.5)	0.030*
No. of swollen joints	0.8 (1.0)	1.1 (1.0)	0.034*
CHAQ score	0.8 (0.5)	1.0 (0.5)	0.043*
Parent global assessment of child’s pain VAS	3.8(1.8)	4.6 (1.8)	0.010*
**ERA-specific characteristics**			
No. of enthesitis†	0.4 (0.9)	1.0 (1.3)	0.003*
Tender entheseal score†	1.5 (1.7)	2.2 (1.8)	0.028*
Overall back pain VAS†, mm	13.7 (17.8)	10.3 (13.6)	0.231
Patrick sign, positive‡, n (%)	55 (58.5)	39 (75.0)	0.046*
Nocturnal back pain VAS†, mm	7.7 (13.4)	8.2 (13.4)	0.831

All values are mean (SD), unless specified otherwise.

CRP, C-reactive protein; ESR, erythrocyte sedimentation rate; PGA, physician’s global assessment; VAS, visual analog scale; CHAQ, Childhood Health Assessment Questionnaire; ERA, enthesitis-related arthritis; LOM, limitation of motion.

^†^mean (standard deviation), between-group comparison: Student’s *t*-test.

^‡^frequency/total, n (%), between-group comparisons: chi-square test.

^§^frequency/total, n (%), between-group comparisons: continuity correction.

*p<0.05 denotes a significant difference between different groups.

### Major symptom of fever

ERA patients had a major symptom of fever at onset that lasted from 1 month to 8 months. Mean duration of fever was 1.6 months. A total of 78.8% of patients had low-grade fever, and 15.4% had moderate-grade fever. Remainder of patients (5.8%) suffered from high-grade fever. Three major types of fever were recorded: “irregular”, “intermittent” and “remittent” (Table 2).

**Table 2:** Clinical features of fever in children with ERA.

**Subjects (n)**	52
**Duration of fever** ^†^, **months**	1.6 (1.4)
**Classification of fever** ^‡^, **n (%)**	
Low-grade	41 (78.8)
Moderate-grade	8 (15.4)
High-grade	3 (5.8)
**Patterns of fever** ^‡^, **n (%)**	
Irregular	48 (92.3)
Intermittent	3 (5.8)
Remittent	1 (1.9)

^†^mean (standard deviation).

^‡^frequency/total, n (%).

### Clinical features of arthritis

Distribution of clinically detected articular involvement at disease onset is shown in Table 3. Upon comparison with the non-fever group, those with fever had a markedly higher prevalence of arthritis that affected five or more joints at disease onset (23.1% *vs*. 8.5%, p = 0.014). Significantly more patients had arthritis of the lower limbs involving the sacroiliac joint, ankle, and calcaneus in the fever group (all p<0.05, Table 3). Out of 146 children, 11 had pain in the lumbar spine, 5 had pain in the dorsal spine, 52 had pain in the hip, and 3 reported pain symptoms in the cervical spine. There was no significant difference in lumbar, dorsal, hip, and cervical involvement between the two groups (all p>0.05, Table 3).

**Table 3:** Clinical distribution of articular involvement at ERA onset.

Joint(s) involved	Non-fever (n = 94)	Fever (n = 52)	p
**Spine** ^†^, **n (%)**			
Sacroiliac	33 (35.1)	31 (59.6)	0.004*
Lumbar	9 (9.6)	2 (3.8)	0.353
Dorsal	4 (4.3)	1 (1.9)	0.790
Cervical	1 (1.1)	2 (3.8)	0.599
Hip	29 (30.9)	23 (44.2)	0.106
**Peripheral** ^†^, **n (%)**			
Knee	38 (40.4)	28 (53.8)	0.119
Ankle	17 (18.1)	21 (40.4)	0.003*
Calcaneus	9 (9.6)	12 (23.1)	0.026*
Elbow	3 (3.2)	1 (1.9)	1.000
Feet, tarsus	5 (5.3)	7 (13.5)	0.161
Shoulder	5 (5.3)	5 (9.6)	0.521
Temporomandibular	0	0	/
Hand, proximal interphalangeal	4 (4.3)	4 (7.7)	0.621
Wrist	2 (2.1)	4 (7.7)	0.235
Involvement of ≥5 joints ^†^, n (%)	8 (8.5)	12 (23.1)	0.014*

^†^frequency/total, n (%), between-group comparisons: chi-square test.

*p<0.05 denotes a significant difference between different groups.

### Extra-articular manifestations

The number of patients who had enthesitis was significantly higher in patients with fever compared with the non-fever group (71.2% *vs*. 23.4%, p<0.05). Topographic distribution of clinically abnormal sites between the fever group and non-fever group was: Achilles tendon, 34.6% *vs*. 9.6%; plantar fascia, 15.4% *vs*. 1.1%; patella (at insertions of the quadriceps and patellar tendons), 11.5% *vs*. 2.1% (all p<0.05, Table 4). All patients underwent blood counts upon hospitalization. Significantly more patients in the fever group showed hypochromic or normochromic anemia than those in the non-fever group (53.8% *vs*. 21.3%, p<0.05). Patients in the fever group had markedly higher mean values of ESR (40.8 (29.0) *vs*. 26.4 (20.1) mm/h), CRP (20.7 (29.5) *vs*. 9.7 (14.3) mg/dL), and platelets (353.2 (101.1) ×10^9^/L *vs*. 275.6 (87.4) ×10^9^/L) than the non-fever group (all p<0.05). White blood cell count of all patients was within the normal range. All patients were negative for RF and ANA. Bone-marrow smear under the microscope was undertaken in only 15 cases in the fever group and showed similar results: bone-marrow cells were proliferous and active and phagocytes could be observed.

**Table 4:** Clinical distribution of enthesitis examined at disease onset.

	Non-fever (n = 94)	Fever (n = 52)	p
**No. of examined enthesitis** ^†^, **n (%)**	22 (23.4)	37 (71.2)	0.000*
**Entheseal insertion sites** ^†^, **n (%)**			
Achilles tendon	9 (9.6)	18 (34.6)	0.000*
Plantar fascia	1 (1.1)	8 (15.4)	0.002*
Patella (at insertions of the quadriceps and patellar tendons)	2 (2.1)	6 (11.5)	0.044*
Hip extensor insertion	0 (0)	0 (0)	/
Greater trochanter	0 (0)	0 (0)	/
Pubis	0 (0)	2 (3.8)	0.242
Tibialis anterior tendon	1 (1.1)	2 (3.8)	0.599
Medial and lateral epicondyles	4 (4.3)	1 (1.9)	0.790

^†^frequency/total, n (%), between-group comparisons: chi-square test.

*p<0.05 denotes a significant difference between different groups.

### Medications

Table 5 summarizes the drug use in the observational study. Non-steroidal anti-inflammatory drugs (NSAIDs) and disease-modifying anti-rheumatic drugs (DMARDs) were the mainstays of treatment. Significantly more patients in the fever group received NSAIDs, corticosteroids, DMARDs, and antibiotic treatment than those in the non-fever group at disease onset (all p<0.05, Table 5). At 6-, 12-, and 24-month follow-up, significantly more patients in the fever group were treated with NSAIDs and corticosteroids (all p<0.05). At the end of follow-up, a DMARD combination (≥2) was employed significantly more often in the fever group than in the non-fever group (p<0.05). A biologic agent co-treated with DMARDs was employed more often in the fever group, but there was no significant difference between the two groups during the study (all p>0.05, Table 5).

**Table 5:** Drug treatment in ERA at onset, 6 months, 12 months, 18 months and end of follow-up.

Time point	Non-fever (n = 94)	Fever (n = 52)	p
**Disease onset** ^†^			
Oral NSAID	67 (71.3)	51 (98.1)	0.000*
DMARD	29 (30.9)	30 (57.7)	0.002*
Oral corticosteroids	3 (3.2)	13 (25.0)	0.000*
Biologics	21 (22.3)	16 (29.6)	0.324
Biologics combined with DMARDs	14 (14.9)	8 (15.4)	0.937
Antibiotic treatment^§^	1 (1.1)	7 (13.5)	0.006*
**6 month follow-up** ^†^			
Oral NSAID	45 (47.9)	41 (78.8)	0.000*
DMARD	72 (76.6)	37 (71.2)	0.469
Oral corticosteroids	6 (6.4)	23 (44.2)	0.000*
Biologics	26 (27.7)	21 (40.4)	0.115
Biologics combined with DMARDs	23 (24.5)	19 (35.8)	0.142
**12 month follow-up** ^†^			
Oral NSAID	23 (24.5)	31 (59.6)	0.000*
DMARD	82 (87.2)	48 (92.3)	0.347
Oral corticosteroids	8 (8.5)	21 (40.4)	0.000*
Biologics	28 (29.8)	17 (32.7)	0.716
Biologics combined with DMARDs	27 (28.7)	17 (32.7)	0.617
**18 month follow-up** ^†^			
Oral NSAID	9 (9.6)	17 (32.7)	0.000*
DMARD	88 (93.6)	51 (98.1)	0.227
Oral corticosteroids	8 (8.5)	19 (36.5)	0.000*
Biologics	31 (33.0)	19 (36.5)	0.664
Biologics combined with DMARDs	31 (33.0)	18 (34.6)	0.841
**End of follow-up**			
Oral NSAID^†^	5 (5.3)	14 (26.9)	0.000*
DMARD^§^	82 (86.3)	52 (100)	0.013*
Oral corticosteroids^†^	6 (6.4)	19 (36.5)	0.000*
Biologics^†^	31 (33.0)	20 (38.5)	0.506
Biologics combined with DMARDs^†^	29 (30.9)	20 (40.4)	0.245
Number of DMARDs taken (≧2)^†^, n (%)	21 (22.3)	32 (61.5)	0.000*

Biologic agents were etanercept and adalimumab.

NSAID, non-steroidal anti-inflammatory drug; DMARD, disease-modifying anti-rheumatic drug.

^†^frequency/total, n (%), between-group comparisons: chi-square test.

^§^ frequency/total, n (%), between-group comparisons: continuity correction.

*p<0.05 denotes a significant difference between different groups.

### Imaging

**Fig 1:**
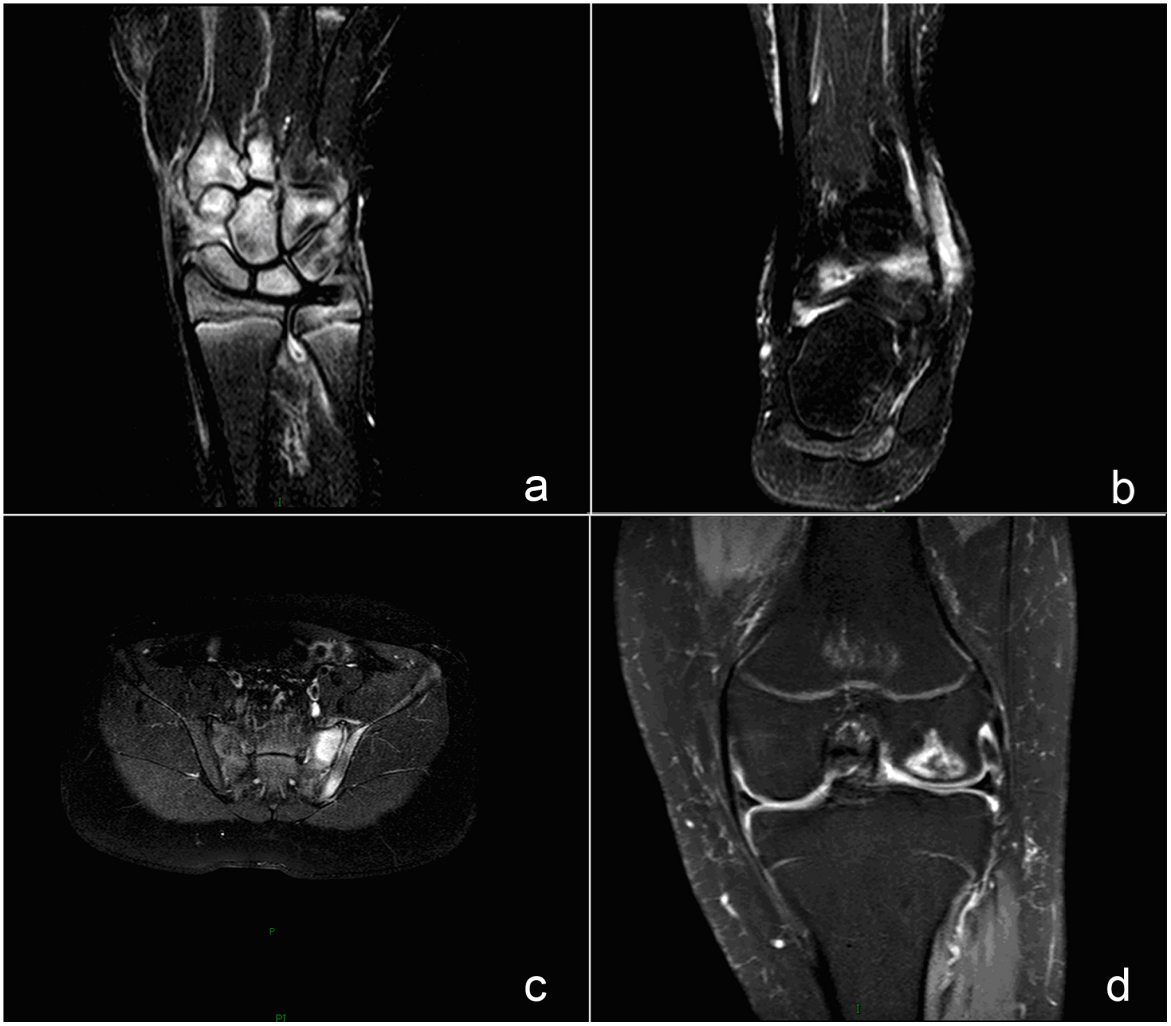
MRI findings of frequently involved joints. (a, b, c and d) Bone marrow edema (BME) appears as hyperintense signal in SPAIR sequence, reflecting active inflammatory lesion. a SPAIR image of wrist demonstrates BME on the carpal. b SPAIR image of a ankle shows BME at the tibiofibula. c SPAIR image of sacroiliac joint shows BME on the left of sacroiliac joint. d SPAIR image of a knee joint shows BME at the distal femur.

According to physical examination, magnetic resonance imaging (MRI) of involved joints was undertaken in all patients without giving gadolinium contrast. MRI findings of the two groups are summarized in Table 6. Bone-marrow edema (BME) is a sign of acute and active arthritis (Fig 1). BME was present in 40/52 in the sacroiliac joint, 13/52 in the tibia, and 14/52 in the knee in the fever group. In the non-fever group, BME was present in 37/94 in the sacroiliac joint, 9/94 in the tibia, and 4/94 in the knee. Comparison between the two groups indicated a significantly higher prevalence of BME of the sacroiliac joint, tibia, and knee in the fever group (all p<0.05, Table 6). Synovitis and effusion of involved joints are also described in Table 6.

**Table 6:** Summary of MRI imaging data of involved joints in ERA patients at disease onset.

Finding	Non-fever (n = 94)	Fever (n = 52)	p
**Bone-marrow edema** ^†^,**n (%)**			
Sacroiliac	37 (39.4)	40 (76.9)	0.000*
Tibia	9 (9.6)	13 (25.0)	0.013*
Feet	7 (7.4)	8 (15.4)	0.130
Knee	4 (4.3)	14 (26.9)	0.000*
Hip	13 (13.8)	11 (21.2)	0.253
Pubis	0 (0.0)	3 (5.8)	0.081
Lumbar	1 (1.1)	1 (1.9)	1.000
Wrist	0 (0.0)	2 (3.8)	0.242
Ankle	5 (5.3)	2 (3.8)	1.000
Coracoid	0 (0.0)	2 (3.8)	0.242
**Synovitis** ^†^,**n (%)**			
Wrist	0 (0.0)	1 (1.9)	0.763
Hip	4 (4.3)	0 (0.0)	0.328
Knee	0 (0.0)	3 (5.8)	0.081
**Joint effusion** ^†^,**n (%)**			
Ankle	2 (2.1)	8 (15.4)	0.007*
Knee	4 (4.3)	12 (23.1)	0.000*
Hip	12 (12.8)	12 (23.1)	0.107
Feet	0 (0.0)	3 (5.8)	0.081
Sacroiliac	1 (1.1)	1 (1.9)	1.000
Wrist	0 (0.0)	2 (3.8)	0.242
Atlantoaxial	0 (0.0)	1 (1.9)	0.763

^†^frequency/total, n (%), between-group comparisons: chi-square test.

*p<0.05 denotes a significant difference between different groups.

### Ultrasonography

**Fig 2:**
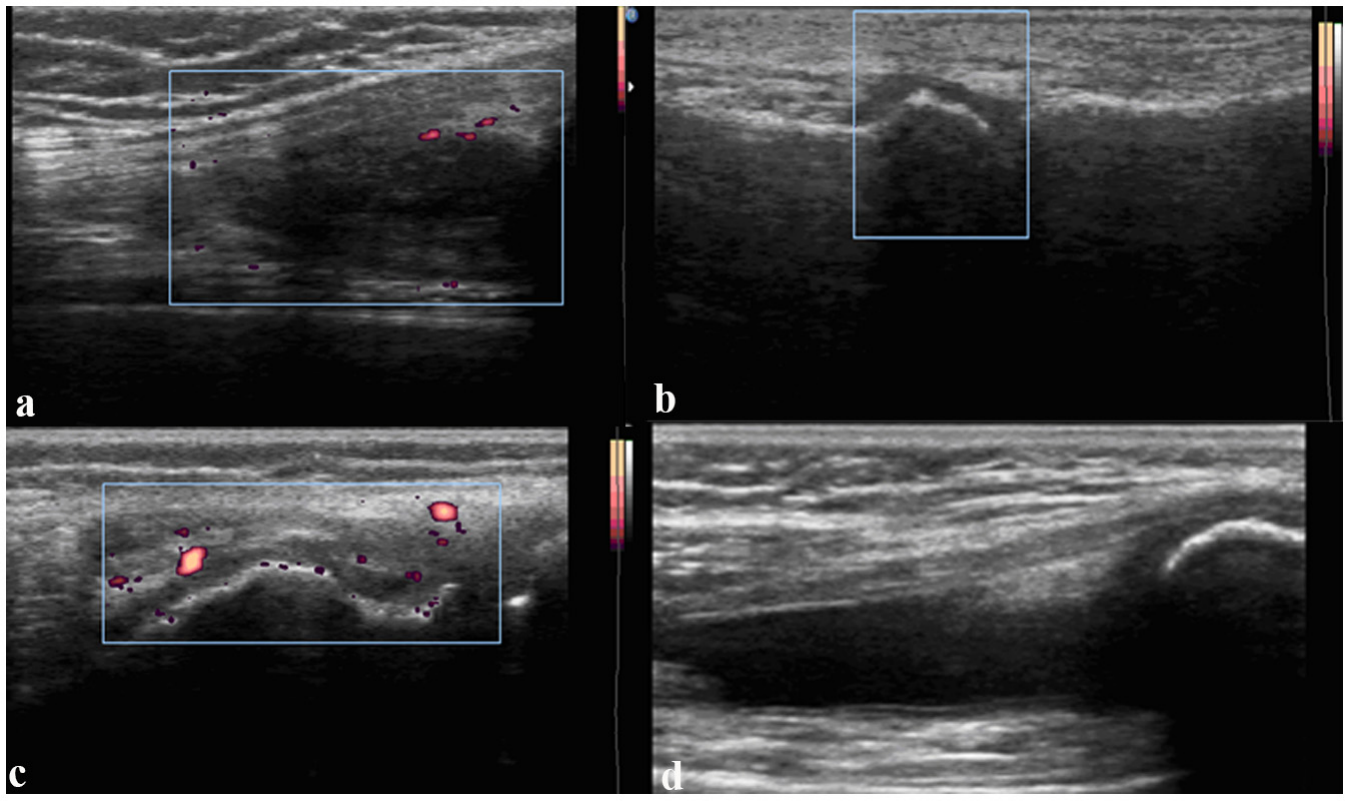
US synovitis of frequently involved joints. a shows longitudinal image of suprapatellar bursa with synovial proliferation, synovial fluid and vascularization. b shows image of the MTP1 with synovial proliferation without synovial fluid and joint vascularization. c demonstrates longitudinal image of lateral femoral condyle of the knee with synovial proliferation and vacularization. d shows longitidudinal image of suprapatellar bursa.

**Fig 3:**
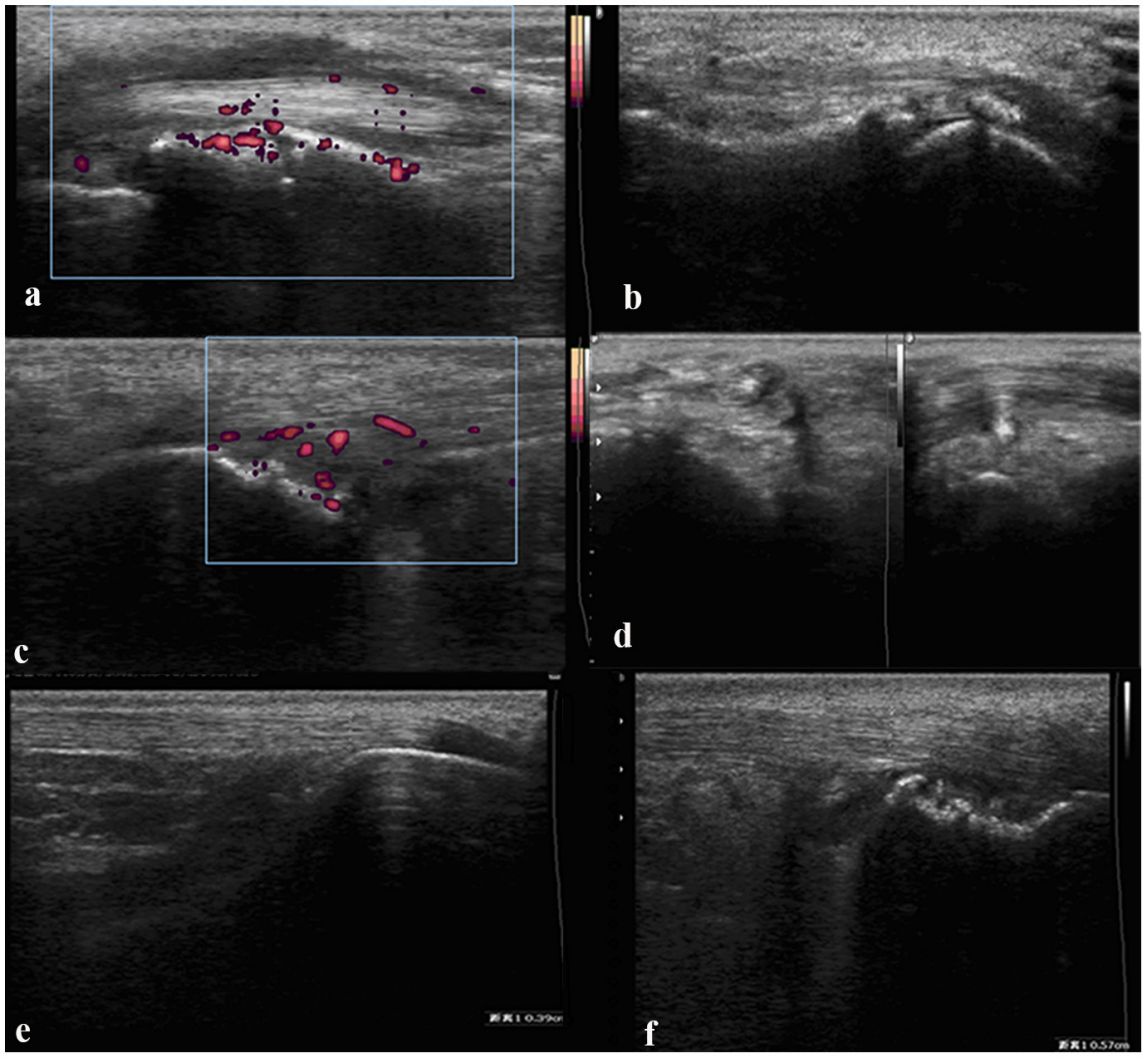
US abnormalitis in entheses. a shows tendon sheath with fluid, vacularization and synovial proliferation. b shows marked calcific insertional tendinopathy. c shows the signal of power doppler which is located inside the calcaneal tendon and tendon thickening. d demonstrates anterior tibial tendon with hypoechogenicity. e and f show the Achilles tendon of the same patient. e demostrates the normal Achilles tendon. f shows tendon thickening and calcaneal erosions.

Ultrasound (US) abnormalities were recorded in 29 patients (20 in the fever group, 9 in the non-fever group). Among them, 18 (62.1%) had US synovitis (defined as synovial hypertrophy with or without synovial fluid and/or joint vascularization; Fig 2) and enthesitis was noted in 13 (44.8%) cases. US abnormalities in enthesitis were enthesophytes, calcifications, tendon thickening, and hypoechogenicity (Fig 3). Most common site of enthesitis in children was calcaneus at the insertion of the Achilles tendon (40%) and at the attachment of the plantar fascia (20%). Tibial tuberosity (patellar tendon), and the anterior surface of the patella (quadriceps tendon) were the other common sites of enthesitis in children (10% for each). Less common sites were the fibular head and ischial tuberosity.

### Follow-up

At the end of two-year follow-up, the number of ERA flares (≧2), number of cases of active disease, and CHAQ score in patients with fever were significantly higher than those of the non-fever group (all p<0.005, Table 7).

**Table 7:** Clinical features of ERA patients at the end of two-year follow-up.

	Non-fever (n = 94)	Fever (n = 52)	p
**Number of cases of active disease** ^†^, **n (%)**	45 (47.9)	41 (78.8)	0.000*
**Number of flares (≧2)** ^†^, **n (%)**	24 (25.5)	25 (48.1)	0.006*
**CHAQ score** ^‡^, **mean (SD)**	0.3 (0.4)	0.5 (0.4)	0.010*

CHAQ, Childhood Health Assessment Questionnaire.

^†^frequency/total, n (%), between-group comparisons: chi-square test.

^‡^mean (standard deviation), between-group comparison: Student’s t-test.

*p<0.05 denotes a significant difference between different groups.

## Discussion

In the present study, fever was a frequent manifestation in ERA. Fever often occurs in response to infection, inflammation and trauma. However, this view of fever is an oversimplification: a growing body of evidence suggests that fever represents a complex adaptive response of the host to various immune challenges, whether infectious or non-infectious [10]. Among 290 patients with chronic joint pain in our study, the prevalence of ERA (50.3%) was significantly higher than the prevalence of ERA reported in western countries (7–13%) [18,19]. The fact that in the present study ERA patients were a homogeneous population and that the epidemiology of JIA in a multiethnic cohort also showed significantly differences in distribution of JIA subtypes among ethnic groups [20,21] could explain the different range of values that have been reported.

ERA with a major symptom of fever at disease onset does not appear to be rare even though exact data of its occurrence are lacking. In the present study, 35.6% of ERA patients had fever as one of the first symptoms at disease onset (which may have been a challenge for the diagnosis) and 13.5% received antibiotics due to suspected infection, which was significantly higher than that observed for the non-fever group (1.1%). Among patients with fever, fever occurred simultaneously in the disease course and lasted for a mean duration of 1.6 months with an irregular pattern. In addition, there was a longer mean interval of 6.7 months in the fever group before patients obtained an accurate diagnosis. A misdiagnosis of septic arthritis and soJIA was made in 10 cases (19.2%) in the fever group, which was higher than that in the non-fever group. There is evidence that extra-articular manifestations and comorbidities (e.g., eye discomfort, skin/nail problems, inflammatory bowel disease, unexplained fever) are common in ankylosing spondylitis (AS) [22] and that comorbidities reduce the quality of life of AS patients [23]. In our study, ERA patients with fever at disease onset had more painful and swollen joints, enthesitis, as well as a high parent global assessment of child’s pain VAS score, parent global assessment of child’s overall well-being, entheseal score, and CHAQ score than the non-fever group. Higher levels of disability are predicted by higher levels of CHAQ at disease onset and higher parent global assessment of child’s overall well-being [24]. These findings suggested that ERA patients with fever had higher disease activity at disease onset than those without fever. Unlike adult spondyloarthritis, inflammatory back pain is rarely present at the onset of ERA [25]. In accordance with that finding, overall back-pain VAS scores and nocturnal back-pain VAS scores were low in both groups (Table 1).

Patients with fever differ in many aspects from patients without fever with regard to clinical manifestations, laboratory tests, imaging data, and medications. Common clinical manifestations of ERA include arthritis, enthesitis, and acute anterior uveitis; axial disease is also common in children with established ERA [2]. Elevated ESR is a marker of disease severity that can also be used to predict the development of sacroiliitis [26]. All patients with fever had a significantly higher ESR, CRP level, platelet count, and had more arthritis (sacroiliac joint, ankle, calcaneus) or enhesitis than the non-fever group. Several epidemiologic studies on AS have found extra-articular manifestations to be a consequence of uncontrolled systemic inflammation [22]. Higher disease activity, elevated levels of inflammatory makers, and fever may also be consequences of uncontrolled systemic inflammation in ERA. Patrick’s test was positive in 75% of the fever group, which was obviously higher than that in the non-fever group (58.5%). This result is different from other studies, which considered tenderness in the sacroiliac joint and/or inflammatory spinal pain to usually occur at a later stage [3]. In our study, ERA onset was usually in late childhood or adolescence, so the age of onset of disease appeared comparatively later with a peak distributed over the pubertal stage. Also, there was a longer delay from the onset of the first symptom to the definitive diagnosis in the fever group. These reasons may explain (at least in part) why many patients had clinical and MRI features of sacroiliitis at disease onset (especially patients in the fever group). Enthesitis had a remarkable predominance for the lower extremities at disease onset, accounting for 71.2% of cases in the fever group. Hence, patients with fever had higher values of inflammatory indicators (ESR, CRP level, platelet count), more sacroiliitis, and more enthesitis than those without fever, which may be not beneficial for disease control [25].

Hypochromic or normochromic anemia is considered to be more frequent in ERA with fever, possibly because most patients are observed at a late stage, have obvious systemic inflammatory features, and suffer from a slowly progressive condition without complete permanent remission [2]. All patients were negative for RF and ANA. This negative result is in accordance with a previous study [2]. A higher proportion of HLA-B27 positivity and family history of HLA-B27-associated disease was shown in the present study. These findings differ from studies conducted on Philadelphia, USA [27], which may be explained by the different ethnicities and regions employed. However, Shen et al. found that HLA-B27-positive patients tended to have a continuously active or relapsing course [1] and longer duration of elevation of blood-inflammation markers compared with HLA-B27-negative patients [28]. Interestingly, we showed that 47 (32.2%) of ERA patients were atopic, which could have been associated with immunologic dysregulation [29].

Due to the absence of exposure to radiation and higher sensitivity compared with conventional radiography, MRI can be used to identify and grade acute and chronic signs of involvement of sacroiliac joints earlier than that by radiography [4]. This feature allowed us to detect acute inflammatory changes in sacroiliac joints in 43.8% of children higher than previous data, which showed that the prevalence of such involvement was homogenous, ranging from 20% to 32% [25]. In addition, the prevalence of BME in sacroiliitis in ERA patients with fever (59.6%) was significantly higher than in the non-fever group (35.1%). Given that MRI of sacroiliac joints was carried out only in patients with a positive Patrick’s test, these findings need further investigation. Furthermore, all patients had MRI of involved joints based on clinical manifestations. These MRI results (Table 6) further confirmed that significantly more patients had arthritis of the lower limbs involving the sacroiliac joint, ankle, and tibia in the fever group.

Dynamic contrast-enhanced MRI is highly sensitive and useful for early detection of joint changes [25]. However, no patients received gadolinium contrast because of the risk of allergic reactions. We showed that 47 (32.2%) of all ERA patients were atopic. Gadolinium contrast might be considered carefully only if treatment effects are suboptimal and US shows obvious synovial hyperplasia. US is a very reliable, safe and relatively inexpensive imaging modality used for the study of joint inflammation [30]. However, US was employed in only 29 patients, all of whom had abnormalities. Most prevalent sites of enthesitis were the: calcaneus at the insertion of the Achilles tendon; attachment of the plantar fascia; tibial tuberosity (patellar tendon); anterior surface of the patella (quadriceps tendon). These findings are in accordance with a US study for the detection of enthesitis in JIA [17]. Enthesitis in ERA with fever was often asymmetric and affected primarily the lower limbs.

Introduction of biologic agents has spawned optimism that treatment will lead to improved outcomes for ERA [15]. Use of biologic compounds in our cohort reflects their steadily increasing use during the past decade. In addition to biologic agents, NSAIDs and DMARDs were the mainstays of treatment. Ultimate determination of treatment is made by the physician in light of individual circumstances. Considering the possible side-effects in juveniles, medications were administered with great care in our study. There was a gradual increase in the number of patients receiving DMARDs, biologics, and biologics combined with DMARDs (Table 5). There were no significant differences in the use of biologics and biologics combined with DMARDs, but a combination of DMARDs (≥2) was employed significantly more often in the fever group at the end of follow-up, thereby suggesting a difference in treatment. ERA patients with fever were treated more aggressively in the use of DMARDs than the non-fever group. Also, patients with fever suffered from systemic inflammatory features such as repeat fever and high values of ESR. Hence, corticosteroids (oral corticosteroid at ≤0.2 or 10 mg/day, p.o.) were administered more frequently in patients with fever than in those without fever. Therefore, treatment of active systemic features (e.g., fever) and active arthritis for ERA patients should be considered separately and independently from ERA patients without systemic features. Significantly more patients in the fever group received NSAIDs at disease onset as well as at 6-, 12-, and 24-month follow-up, which might suggest that pain relief in the fever group was relatively slow. Moreover, at disease onset, 13.5% of the fever group received antibiotic therapy because of suspected infection, whereas only 1.1% of patients without fever were treated with antibiotics. A similar study also showed that 46% of patients with juvenile SpA had fever and received antibiotic therapy, which was higher than in our study [30]. This high prevalence of subjects receiving antibiotic therapy might be related to the small group sizes.

To determine outcomes in patients with fever, at the end of two-year follow-up, we analyzed the CHAQ score, number of disease flares, and number of cases of active disease. Significantly more patients in the fever group had a high CHAQ score, number of disease flares, and active disease. This is the first time that this phenomenon has been reported, and suggests that ERA patients with fever might have more severe disease and poorer outcomes than ERA patients without fever.

The present study had limitations. It was a retrospective analysis with a relatively small cohort. Also, we could not retrieve all requisite findings (primarily the utility of US in enthesitis).

## Conclusions

This was the first study to compare the clinical manifestations, treatment course, as well as the laboratory and imaging features of ERA patients with fever to those without fever in a systematic manner. Our results provide a good starting point to study ERA patients with systemic features (e.g., fever, anemia, leukocytosis) as the main manifestations at disease onset.
